# Clinical Factors Associated with Cavitary Tuberculosis and Its Treatment Outcomes

**DOI:** 10.3390/jpm11111081

**Published:** 2021-10-25

**Authors:** Sun-Hyung Kim, Yoon Mi Shin, Jin Young Yoo, Jun Yeun Cho, Hyeran Kang, Hyun Lee, Kang Hyeon Choe, Ki Man Lee, Bumhee Yang

**Affiliations:** 1 Division of Pulmonary and Critical Care Medicine, Department of Internal Medicine, Chungbuk National University Hospital, Chungbuk National University College of Medicine, Cheongju 28644, Korea; iinakaii79@gmail.com (S.-H.K.); anees94@hanmail.net (Y.M.S.); ok_kaist2115@hanmail.net (J.Y.C.); hr830901@naver.com (H.K.); choekh@chungbuk.ac.kr (K.H.C.); kimlee@chungbuk.ac.kr (K.M.L.); 2 Department of Radiology, Chungbuk National University Hospital, Chungbuk National University College of Medicine, Cheongju 28644, Korea; dranne@naver.com; 3 Division of Pulmonary Medicine and Allergy, Department of Internal Medicine, Hanyang University College of Medicine, Seoul 04763, Korea; namuhanayeyo@hanyang.ac.kr

**Keywords:** tuberculosis, cavity, association factors, treatment outcomes

## Abstract

Cavitary pulmonary tuberculosis (TB) is associated with poor outcomes, treatment recurrence, higher transmission rates, and the development of drug resistance. However, reports on its clinical characteristics, associated factors, and treatment outcomes are lacking. Hence, this study sought to evaluate the clinical factors associated with cavitary pulmonary TB and its treatment outcomes. We retrospectively evaluated 410 patients with drug-susceptible pulmonary TB in a university hospital in Korea between 2014 and 2019. To evaluate the factors associated with cavitary TB, multivariable logistic regression was performed with adjustments for potential confounders. We also compared the treatment outcomes between patients with cavitary TB and those without cavitary TB. Of the 410 patients, 244 (59.5%) had non-cavitary TB and 166 (40.5%) had cavitary TB. Multivariable logistic analysis with forward selection method showed that body mass index (BMI) (adjusted OR = 0.88, 95% CI: 0.81–0.97), previous history of TB (adjusted OR = 3.45, 95% CI: 1.24–9.59), ex- or current smoker (adjusted OR = 1.77, 95% CI: 1.01–3.13), diabetes mellitus (adjusted OR = 2.72, 95% CI: 1.36–5.44), and positive results on the initial sputum acid-fast bacilli (AFB) smear (adjusted OR = 2.24, 95% CI: 1.26–3.98) were significantly associated with cavitary TB. Although treatment duration was significantly longer in patients with cavitary TB than in those with non-cavitary TB (248 (102–370 days) vs. 202 (98–336 days), *p* < 0.001), the recurrence rate after successful treatment was significantly higher in the patients with cavitary TB than in those with non-cavitary TB (0.4% vs. 3.0% *p* = 0.042). In conclusion, ex- or current smoker, lower BMI, previous history of TB, diabetes mellitus, and positivity of the initial AFB smear were associated with cavitary TB. The patients with cavitary TB had more AFB culture-positive results at 2 months, longer treatment duration, and higher recurrence rates than those with non-cavitary TB.

## 1. Introduction

The proportion of patients with pulmonary tuberculosis (TB) who have a cavitary disease at the time of diagnosis ranges from 29 to 87% [1,2,3]. Several studies have reported that the high oxygen concentration inside the cavity facilitates bacterial replication and increases the bacterial loads in the sputum [4,5,6]. In addition, anti-TB drugs cannot penetrate the cavity easily [7,8], leading to treatment failure or disease recurrence [3,9,10,11,12]. Hence, cavitary disease is associated with high bacterial load, treatment failure, and recurrence.

Since patients with cavity TB have a severe presentation and poor treatment outcomes, understanding the factors associated with cavitary TB could provide useful information to clinicians. Previous studies showed that diabetes mellitus (DM), alcohol abuse, and smoking history were associated with cavitary TB [13,14,15,16,17]. However, the primary study outcomes of previous studies were not to evaluate factors associated with cavity TB. For example, the association between DM and cavity TB was revealed while evaluating the clinical factors associated with diabetic tuberculosis [15,18,19]. Similarly, the association between cavitary TB and alcohol was derived from a study evaluating clinical characteristics of multi-drug resistant (MDR-TB) [16,17]. The relationship between cavitary TB and smoking was found in a study evaluating the impact of smoking on the treatment outcomes of pulmonary TB.

Accordingly, to date, there is a paucity of studies that comprehensively evaluate clinical factors associated with cavitary TB, and studies comparing the clinical factors of pulmonary TB according to the presence or absence of cavitary lesions are necessary.

## 2. Material and Methods

### 2.1. Patients

**Figure 1:**
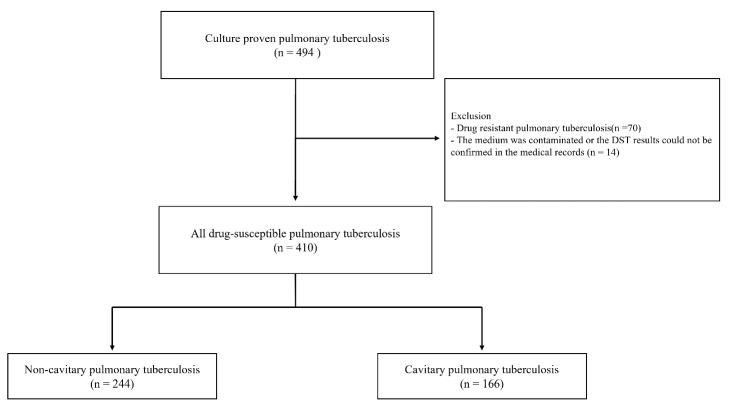
Flow chart of the study. DST, Drug-susceptible test.

We retrospectively reviewed the medical records of 494 consecutive patients with a culture-proven diagnosis of pulmonary TB at Chungbuk National University Hospital (a 793-bed referral hospital in Cheongju, South Korea) between January 2014 and December 2019. Of these 494 patients, those with drug-resistant TB (*n* = 70), those who had their media contaminated, or those who had drug-susceptible test (DST) results that could not be confirmed in the medical records (*n* = 14), were excluded (Figure 1). A total of 410 patients were included in the study approved by the Institutional Review Board of Chungbuk National University Hospital (IRB No. 2020-12-020). Patient information was anonymized and deidentified before the analysis. Therefore, the need for informed consent was waived.

### 2.2. Microbiologic and Radiologic Examination

Sputum acid-fast bacilli (AFB) smears and cultures were performed using standard methods [20]. For smear-positive specimens, the results were graded as ≥1+. All specimens were cultured on a solid medium (Becton-Dickinson and Co., Sparks, MD, USA) and in a liquid broth medium (3% Ogawa agar Shinyang, Seoul, Korea) in mycobacterial growth indicator tubes. Pulmonary TB was diagnosed based on sputum AFB culture or nucleic acid amplification test (NAAT): real-time PCR (PNAqPCR™ TB/NTM detection kit; PANAGENE, Daejeon, Korea), or Xpert MTB/RIF (GeneXpert^®^ System, Cepheid, Sunnyvale, CA, USA). When TB was confirmed by biopsy, the pathologist diagnosed TB, or the tissue was positive for real-time PCR (PNAqPCR™ TB/NTM detection kit; PANAGENE, Daejeon, Korea). Conventional DST was performed using the absolute concentration method with Löwenstein–Jensen media at the Korean Institute of Tuberculosis [21]. Follow-up sputum smear and culture were conducted at 2, 4, and 6 months, and at the end of the TB treatment. Chest X-ray was available for all the patients at the time of diagnosis of TB. The presence of cavitation was determined by three authors: two pulmonologists (S.-H.K. and B.Y.), and one radiologist (J.Y.Y.), after interpreting the chest X-rays. These authors also examined the chest X-rays of the patients with cavitary TB for bilateral involvement, cavity number, and cavity size (the largest size was measured in patients with 2 or more cavities), and a consensus was achieved.

### 2.3. Anti-TB Drugs

The standard treatment for pulmonary TB involves an intensive phase for 2 months and a continuation phase for 4 months [22,23]. The daily regimen for the intensive phase comprises rifampin, isoniazid, ethambutol, and pyrazinamide, and the daily regimen for the continuation phase comprises rifampin and isoniazid with or without ethambutol. For patients with symptoms associated with a high risk for recurrence, including cavitation on chest X-ray and a positive culture after 2 months, the continuation phase was extended to seven months, corresponding to nine months of treatment. The continuation phase can also be extended based on the expectation of an unfavorable outcome based on the discretion of the attending physicians.

### 2.4. Outcomes

The treatment outcomes were classified as “cured,” “treatment completion,” “treatment failure,” “loss to follow-up,” “not evaluated,” and “recurrence” [24,25]. A pulmonary TB patient was one who became smear- or culture-negative within the last month of treatment and, on at least one previous occasion, was classified as cured. A TB patient who received complete treatment was treated without evidence of failure but had no record to show that the sputum smear or culture results within the last month of treatment, and on at least one previous occasion, were negative because tests were not performed or the results were unavailable. Treatment failure was defined as positive sputum smear or culture results after 4 months during treatment. Loss-to-follow-up was defined as not initiating treatment or interrupting it for 2 consecutive months or more. Non-evaluation was defined as the non-assignment of a treatment outcome, such as death during treatment. We further defined a favorable outcome as being cured or completing treatment with no evidence of recurrence during the follow-up. Unfavorable outcomes included treatment failure and recurrence after initial treatment success. The recurrence was defined as positivity of the AFB culture or radiographical deterioration within a year for patients who completed treatment. The patients were requested to visit the hospital every 3 months for at least 1 year after treatment completion for assessment of recurrence.

### 2.5. Statistical Analysis

The data are presented as the mean and standard deviation (SD) for continuous variables and frequency (percentage) for categorical variables. The data were compared using the Student’s *t*-test for continuous variables and the chi-squared test or Fisher’s exact test for categorical variables. A multiple binary logistic regression analysis with forward stepwise selection with *p* < 0.05 for entry of variables and *p* > 0.05 for removal of a variable. Initial candidate variables were age, sex, body mass index (BMI), previous history of TB, smoking history, diabetes mellitus, initial AFB smear, NAAT, and bilateral lung involvement on chest X-ray. Variables selected in the final model were body mass index (BMI), previous history of TB, smoking history, diabetes mellitus, and the initial AFB smear. All tests were two-sided, and *p*-values of <0.05 represented statistical significance. All statistical analyses were performed using IBM SPSS Statistics for Windows (version 21.0; IBM Corp., Armonk, NY, USA) and STATA (version 15; Stata Corp., College Station, TX, USA).

## 3. Results

### 3.1. Baseline Characteristics

The baseline characteristics of the 410 patients with pulmonary TB are shown in Table 1. The mean age of the 410 patients was 62 years (SD, 44–80 years), 253 patients (61.7%) were male, and 41 patients (10.0%) had a history of TB. Among the 410 patients, 244 (59.5%) had non-cavitary TB, and 166 (40.5%) had cavitary TB. The patients with cavitary TB, compared with those with non-cavitary TB, were younger (mean 65 years vs. 57 years, *p* < 0.001) and predominantly male (69.3% vs. 56.6%, *p* = 0.010). They also, more commonly, had a history of TB (15.7% vs. 6.1%, *p* = 0.002) and had lower BMI (mean 21.0 kg/m^2^ vs. 22.3 kg/m^2^, *p* < 0.001). The proportion of current smokers was also significantly higher among the patients with cavitary TB than among those with non-cavitary TB (34.0% vs. 16.7, *p* < 0.001). Regarding comorbidities, DM was more common in the patients with cavitary TB than in those with non-cavitary TB (13.5% vs. 24.7%, *p* = 0.004). Patients with cavitary TB showed significantly higher baseline AFB smear positivity (*p* < 0.001) and higher NAAT positivity (*p* = 0.004) rates than those with non-cavitary TB. In addition, bilateral involvement was seen more commonly in the chest X-rays of the patients with cavitary TB than in those with non-cavitary TB (58.4% vs. 43.4%, *p* = 0.003).

### 3.2. Factors Associated with Cavitary TB

Univariable analysis showed that age (unadjusted odds ratio, OR = 0.97, 95% CI: 0.96–0.99), male sex (unadjusted OR = 0.58, 95% CI: 0.38–0.88), BMI (unadjusted OR = 0.90, 95% CI: 0.84–0.96), previous history of TB (unadjusted OR = 2.84, 95% CI: 1.45–5.54), smoking (unadjusted OR = 1.82, 95% CI: 1.20–2.78), diabetes mellitus (unadjusted OR = 2.01, 95% CI: 1.45–5.54), positive results of initial AFB smear (unadjusted OR = 3.39, 95% CI: 2.23–5.16), and bilateral involvement on chest X-ray (unadjusted OR = 1.83, 95% CI: 1.23–2.73) were significantly associated with cavitary TB (Table 2).

Multivariable analysis showed that BMI (adjusted OR = 0.88, 95% CI: 0.81–0.97), previous history of TB (adjusted OR = 3.45, 95% CI: 1.24–9.59), smoking (adjusted OR = 1.77, 95% CI: 1.01–3.13), diabetes mellitus (adjusted OR = 2.72, 95% CI:1.36–5.44), and positive results of initial AFB smear (adjusted OR = 2.24, 95% CI:1.26–3.98) were significantly associated with cavitary TB after adjusting for age, sex, BMI, previous history of TB, smoking, comorbidities, initial AFB smear, NAAT, and bilateral involvement on chest X-ray (Table 2). Initial chest X-rays of several patients with cavities are provided in Supplemental Figure S1.

### 3.3. Anti-TB Drugs

The treatment regimen and duration for culture-proven pulmonary TB are summarized in Table 3. There were no significant differences in the use of rifampin, isoniazid, ethambutol, and pyrazinamide among the anti-TB drug regimens. Of the secondary drugs, the use of injectable drugs and p-aminosalicylic acid was significantly higher in the cavitary TB than in the non-cavitary TB group (6.2% vs. 1.2%, *p* = 0.002, 2.4% vs. 0, *p* = 0.026). Rifampin, isoniazid, and ethambutol were used significantly longer by the cavitary TB patients than the non-cavitary TB patients (218 (124–312 days) vs. 182 (90–274 days), *p* < 0.001; 228 (125–331 days) vs. 188 (94–282 days), *p* < 0.001; 155 (58–252 days) vs. 128 (33–223 days), *p* = 0.005).

### 3.4. Treatment Outcomes

As shown in Table 4, the 2-month AFB culture positivity rate was significantly higher for the patients with cavitary TB than for those with non-cavitary TB (1.6% vs. 4.8%, *p* = 0.030). The mean duration of treatment of all the patients was 236 days (102–370 days), and the treatment lasted significantly longer for the patients with cavitary TB than for those with non-cavitary TB (248 (102–370 days) vs. 202 (98–336 days), *p* < 0.001). Although the rate of favorable treatment outcomes was not significantly different between the patients with cavitary TB and those without cavitary TB, and the recurrence rate after treatment success was significantly higher for the patients with cavitary TB than for those with non-cavitary TB (0.4% vs. 3.0%, *p* = 0.042).

## 4. Discussion

This study evaluated the clinical characteristics, associated factors, and treatment outcomes for drug-susceptible cavitary TB. Multivariable analysis showed that previous or current smoking, lower BMI, previous history of TB, diabetes mellitus, and positivity of the initial AFB smear were associated with a cavitary TB. Regarding the treatment outcomes, despite a longer duration of treatment, patients with cavitary TB had higher recurrence rates than those without cavitary TB.

Although previous studies have revealed several factors associated with cavitary TB, the primary goal of those studies was not focused on this issue. Most factors were revealed secondarily in studies aimed at other subjects. Thus, the significant advantage of this study was to evaluate factors associated with cavitary TB as the primary purpose of our study. This study showed that smoking history and lower BMI were associated with cavitary TB. Cavity formation in TB patients is influenced by the immunity of the host [26]. Cavity formation in patients with TB is associated with the predominance of T-helper (Th)2 CD4+ cells in the alveoli [26,27,28]. This is one of the adaptive immune cells affected by smoking [29], which may explain the association of smoking with cavitary TB. Likewise, lower BMI indicates the presence of a nutritional deficiency and is related to increased pulmonary inflammation and the free neutrophil elastase activity in the lungs [30,31]. This has been suggested to underlie the association between poor nutritional status and severe lung disease. Numerous conditions associated with the lower BMI, such as chronic energy deficiency, frequent pulmonary inflammation, increased oxidative stress, and altered body composition, may contribute to the development of a cavity in pulmonary TB. Further research is warranted to elucidate the exact mechanisms underlying the association between the lower BMI and the development of cavitary pulmonary TB.

Another factor associated with cavitary TB was previous pulmonary TB and diabetes mellitus. Several published comparative studies have revealed that cavitary disease occurs more frequently in patients with diabetes mellitus than in those without [15,18,19,32]. Consistent with these results, our study showed that diabetes mellitus is associated with cavitary TB. In addition, it was also found that previous history of TB is associated with cavitary TB. There are few reports on the association of TB history with cavitary TB. Only a few studies have reported the formation of pulmonary cavities secondary to TB granuloma [33], and further studies are needed in this regard. Positivity of the initial AFB smear was also associated with cavitary TB. Several studies have shown that patients with cavitary TB have higher mycobacterial loads in their sputum [1,5,6,34,35], which may result in the positivity of the initial AFB smear.

Our study showed that 3% of patients with cavitary TB had a relapse after treatment success. The reported rates of relapse after standard 6-month treatment for drug-susceptible pulmonary TB have ranged from 1% to 2% [9]. However, patients with cavitary TB who received standard 6-month treatment have high relapse rates of up to 18% due to a higher mycobacterial load in their sputum [11]. Compared to previous studies, our study showed a lower recurrence rate, which can be explained by the following: In our study, patients with cavitary TB received treatment for a mean of 248 days, which was longer than the standard treatment duration. A 6-month regimen is the current worldwide standard therapy for drug-susceptible pulmonary TB; however, the treatment duration may be extended based on the discretion of the physicians in private-sector practice in Korea. In addition, the public–private mix collaboration policy of the Korean government [36], which has been in place since 2011, may have an additional role in decreasing the recurrence rate by enhancing medication adherence. Nevertheless, compared to patients with non-cavitary TB, patients with cavitary TB had higher recurrence rates. Therefore, more efforts are required for optimal management and prevention of recurrence in patients with cavitary TB.

This study has several limitations. First, it was retrospective in nature and confined to a single center. The sample size was relatively small. Second, the AFB smear and culture tests were not performed at the time of treatment or termination. Thus, our treatment outcome was “complete” for several cases, and several AFB culture results were classified as “not evaluated.” Third, because genotyping was not available, this study could not discriminate relapse as reactivation from recurrence due to exogenous reinfection [37].

## 5. Conclusions

The factors such as smoking history, lower BMI, previous history of TB, and diabetes mellitus, were associated with cavitary TB. Furthermore, cavitary TB was more likely to have a positive initial AFB smear compared to non-cavitary TB. In terms of the treatment outcomes, patients with cavitary TB showed higher AFB culture positivity at 2 months, longer treatment duration, and higher recurrence rates than those with non-cavitary TB. Considering the unfavorable outcomes of cavitary TB, our study suggests the need for appropriate control of the relevant factors affecting cavity formation during the management of TB patients.

## Figures and Tables

**Table 1:** Baseline characteristics of study patients.

Characteristics	All Patients (*n* = 410)	Non-Cavitary TB (*n* = 244)	Cavitary TB (*n* = 166)	*p*-Value
Age, years	62 (44–80)	65 (47–83)	57 (39–75)	<0.001
Sex, male	253 (61.7)	138 (56.6)	115 (69.3)	0.010
Previous history of TB	41 (10.0)	15 (6.1)	26 (15.7)	0.002
BMI (kg/m^2^)	21.8 (18.2–25.4)	22.3 (18.5–26.1)	21.0 (18.0–24.0)	<0.001
Smoking				<0.001
Current smoker	87 (21.2)	37 (16.7)	50 (34.0)	
Ex-smoker	78 (19.0)	49 (22.2)	29 (19.7)	
Never smoker	203 (49.5)	135 (61.1)	68 (46.3)	
Comorbidities				
Diabetes Mellitus	74 (18.0)	33 (13.5)	41 (24.7)	0.004
Cardiovascular disease	53 (12.9)	38 (15.6)	15 (9.0)	0.053
Neurologic disease	36 (8.8)	24 (9.8)	12 (7.2)	0.360
COPD/Asthma	43 (10.5)	26 (10.7)	17 (10.2)	0.893
Chronic kidney disease	12 (2.9)	9 (3.7)	3 (1.8)	0.375
Chronic liver disease	16 (3.9)	8 (3.3)	8 (4.8)	0.429
HIV	3 (0.7)	3 (1.2)	0	0.275
Initial AFB smear				<0.001
Negative	259 (63.2)	182 (74.6)	77 (46.4)	
Positive	151 (36.8)	62 (25.4)	89 (53.6)	
NAAT ^a^				<0.004
Negative	99 (24.1)	73 (29.9)	26 (15.7)	
Positive	223 (54.4)	122 (50.0)	101 (60.8)	
Not performed	88 (21.5)	49 (20.1)	39 (23.5)	
Bilateral involvement	203 (49.5)	106 (43.4)	97 (58.4)	0.003

The data are presented as either mean and standard deviation, or number and percentage, as appropriate. ^a^ NAAT positivity was characterized by the real-time PCR or Xpert MTB/RIF positivity. TB, Tuberculosis; BMI, body mass index; COPD, chronic obstructive lung disease; HIV, human immunodeficiency virus; AFB, acid-fast bacilli; NAAT, nucleic acid amplification test.

**Table 2:** Factors associated with cavitary TB.

Variable	Unadjusted		Multivariate Analysis
	Unadjusted OR	*p*-Value	Adjusted OR * (95% CI)
Age	0.97 (0.96–0.99)	<0.001	
Male	0.58 (0.38–0.88)	0.010	
BMI, kg/m^2^	0.90 (0.84–0.96)	0.002	0.88 (0.81–0.97)
Previous history of TB	2.84 (1.45–5.54)	0.002	3.45 (1.24–9.59)
Smoking history			
Never-smoker	Ref.		Ref.
Ex- or current smoker	1.82 (1.20–2.78)	0.005	1.77 (1.01–3.13)
Comorbidities			
Diabetes Mellitus	2.01 (1.26–3.49)	0.004	2.72 (1.36–5.44)
Cardiovascular disease	0.54 (0.29–1.02)	0.055	
Neurologic disease	0.71 (0.35–1.47)	0.362	
COPD/Asthma	0.96 (0.50–1.83)	0.893	
Chronic kidney disease	0.48 (0.13–1.80)	0.277	
Chronic liver disease	1.49 (0.55–4.06)	0.432	
Initial AFB smear			
Negative	Ref		Ref
Positive	3.39 (2.23–5.16)	<0.001	2.24 (1.26–3.98)
NAAT			
Negative	Ref		
Positive	2.32 (1.38–3.91)	0.001	
Bilateral involvement	1.83 (1.23–2.73)	0.003	

The data are presented as a ratio (95% CI) * A multiple binary logistic regression analysis with forward stepwise selection with *p*  <  0.05 for entry of variables and *p*  >  0.05 for removal of a variable. Initial candidate variables were age, sex, body mass index (BMI), previous history of TB, smoking history, diabetes mellitus, initial AFB smear, NAAT, and bilateral lung involvement on chest X-ray. Variables selected in the final model were body mass index BMI, previous history of TB, smoking history, diabetes mellitus, initial AFB smear. OR, Odds ratio; CI, confidence interval; Ref. reference; BMI, body mass index; TB, tuberculosis; COPD, chronic obstructive pulmonary disease; NAAT, nucleic acid amplification test.

**Table 3:** Treatment regimens and durations for patients with cavitary and non-cavitary TB.

	All Patients (*n* = 410)	Non-Cavitary TB (*n* = 244)	Cavitary TB (*n* = 166)	*p*-Value
Regimen of anti-TB drugs				
Rifampin	402 (98.0)	237 (97.1)	165 (99.4)	0.150
Isoniazid	401 (97.8)	237 (97.1)	164 (98.8)	0.322
Ethambutol	393 (95.9)	230 (94.3)	163 (98.2)	0.075
Pyrazinamide	379 (92.4)	222 (91.0)	157 (94.6)	0.177
Fluoroquinolone	88 (21.5)	53 (21.7)	35 (21.1)	0.877
Cycloserine	21 (5.1)	10 (4.1)	11 (6.6)	0.254
Injectable drug	15 (3.7)	3 (1.2)	12 (7.2)	0.002
Prothionamide	8 (2.0)	2 (0.8)	6 (3.6)	0.066
p-aminosalicylic acid	4 (1.0)	-	4 (2.4)	0.026
Linezolid	1 (0.2)	1 (0.4)	0 (0)	1.000
Duration of anti-TB drugs				
Rifampin	196 (101–291)	182 (90–274)	218 (124–312)	<0.001
Isoniazid	204 (104–304)	188 (94–282)	228 (125–331)	<0.001
Ethambutol	139 (43–235)	128 (33–223)	155 (58–252)	0.005
Pyrazinamide	77 (0–157)	71 (4–138)	86 (0–182)	0.093
Fluoroquinolone	34 (0–128)	27 (0–100)	43 (0–160)	0.122
Cycloserine	8 (0–63)	4 (0–35)	14 (0–92)	0.144
Injectable drug	1 (0–9)	0 (0–2)	3 (0–16)	0.011
Prothionamide	2 (0–36)	0 (0–3)	5 (0–59)	0.255
p-aminosalicylic acid	3 (0–40)	-	8 (0–66)	0.087
Linezolid	0 (0–3)	0 (0–3)	-	0.410
Treatment duration, days	236 (102–370)	202 (98–336)	248 (134–392)	<0.001

Data are presented as mean and standard deviation or number and percentage, as appropriate. TB, tuberculosis.

**Table 4:** Treatment outcomes for all patients with cavitary and non-cavitary TB.

Outcomes	All Patients (*n* = 410)	Non-Cavitary TB (*n* = 244)	Cavitary TB (*n* = 166)	*p*-Value
AFB culture results at month 2				<0.030
Positive	12 (2.9)	4 (1.6)	8 (4.8)	
Negative	166 (40.5)	93 (38.1)	73 (44.0)	
Not performed	232 (56.6)	147 (60.2)	85 (51.2)	
Treatment duration, days	236 (102–370)	202 (98–336)	248 (134–392)	<0.001
Treatment outcomes				0.062
Cured/treatment completed	385 (93.9)	204 (83.7)	151 (91.0)	
Treatment failure	0 (0)	0 (0)	0 (0)	
Lost to follow up	11 (2.7)	7 (2.9)	4 (2.4)	
Not evaluated	44 (10.7)	33 (13.5)	11 (6.6)	
Recurrence after treatment success	6 (1.5)	1 (0.4)	5 (3.0)	0.042
Favorable treatment outcomes	348 (84.9)	203 (83.2)	145 (87.3)	0.249
Unfavorable treatment outcomes	6 (1.5)	1 (0.4)	5 (3.0)	0.042

The data are presented as mean and standard deviation or number and percentage, as appropriate. AFB: acid-fast bacilli; TB: tuberculosis.

## Data Availability

The data presented in this study are available on request from the corresponding author.

## Supplementary Materials

The following are available online at https://www.mdpi.com/article/10.3390/jpm11111081/s1. Supplementary Figure S1. Initial chest X-ray and associated factor with cavitary TB patients; (A) 49-year-old male patient.; BMI:21.5, previous smoker, positivity of initial AFB smear(+), Diabetes Mellitus(+), previous history of TB(+); (B) 34-year-old male patient.; BMI:17.3, ex-smoker, positivity of initial AFB smear (+), Diabetes Mellitus (+), previous history of TB (−); (C) 43-year-old male patient; BMI: 16.0, current smoker, positivity of initial AFB smear (+), Diabetes Mellitus (+) previous history of TB (−); BMI: body mass index; AFB: acid-fast bacilli; TB: tuberculosis.
