# Supplementary figures and images for: Clinical Factors Associated with Cavitary Tuberculosis and Its Treatment Outcomes
Source: J Pers Med. 2021 Oct 25;11(11):1081. doi: 10.3390/jpm11111081 (PMC8622689; doi:10.3390/jpm11111081)

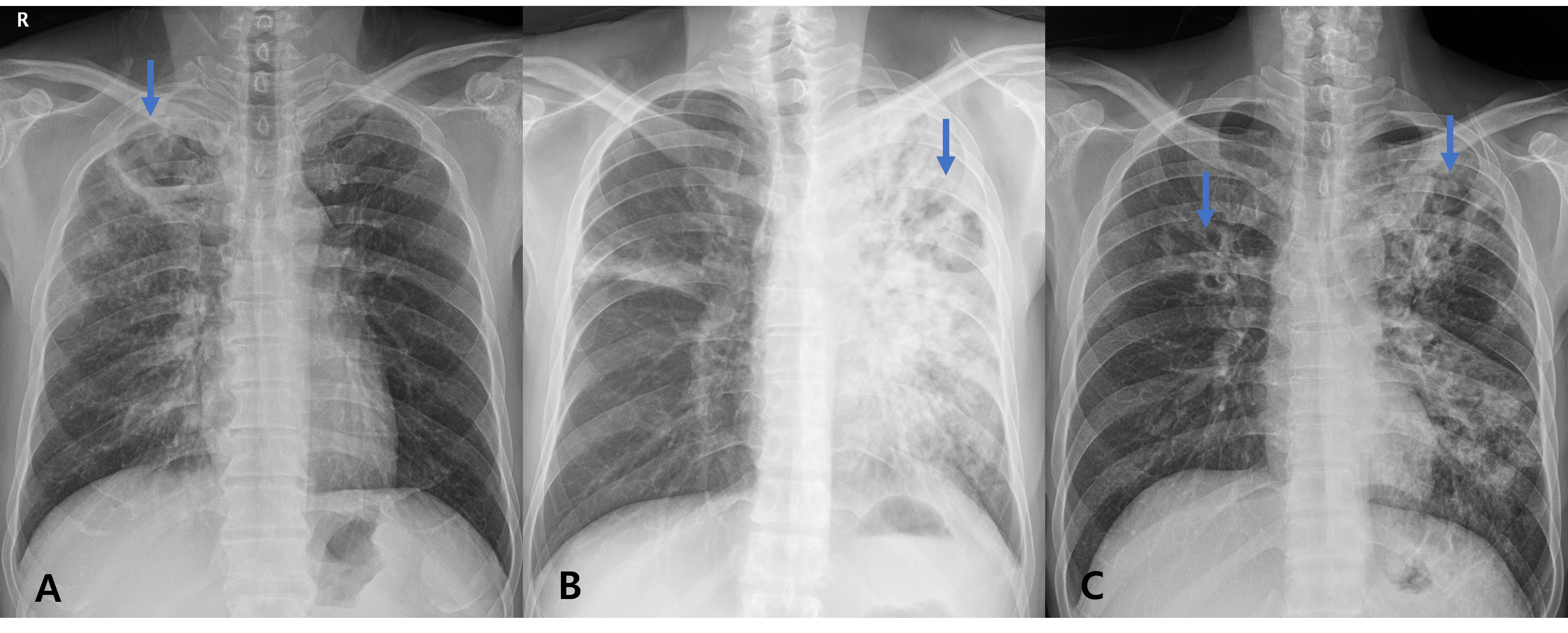

Supplement: Supplementary file 1 [file jpm-11-01081-s001.zip › jpm-1346814-Figure S1.png]
